# An in vivo model for extracellular vesicle–induced emphysema

**DOI:** 10.1172/jci.insight.153560

**Published:** 2022-01-25

**Authors:** Camilla Margaroli, Matthew C. Madison, Liliana Viera, Derek W. Russell, Amit Gaggar, Kristopher R. Genschmer, J. Edwin Blalock

**Affiliations:** 1 Department of Medicine, Division of Pulmonary, Allergy and Critical Care Medicine,; 2 Program in Protease and Matrix Biology, and; 3 Lung Health Center and Gregory Fleming James Cystic Fibrosis Center, University of Alabama at Birmingham, Birmingham, Alabama, USA.; 4 Birmingham VA Medical Center, Birmingham, Alabama, USA.

**Keywords:** Pulmonology, COPD, Extracellular matrix, Neutrophils

## Abstract

Chronic obstructive pulmonary disease (COPD) is a debilitating chronic disease and the third-leading cause of mortality worldwide. It is characterized by airway neutrophilia, promoting tissue injury through release of toxic mediators and proteases. Recently, it has been shown that neutrophil-derived extracellular vesicles (EVs) from lungs of patients with COPD can cause a neutrophil elastase–dependent (NE-dependent) COPD-like disease upon transfer to mouse airways. However, in vivo preclinical models elucidating the impact of EVs on disease are lacking, delaying opportunities for therapeutic testing. Here, we developed an in vivo preclinical mouse model of lung EV–induced COPD. EVs from in vivo LPS-activated mouse neutrophils induced COPD-like disease in naive recipients through an α-1 antitrypsin–resistant, NE-dependent mechanism. Together, these results show a key pathogenic and mechanistic role for neutrophil-derived EVs in a mouse model of COPD. Broadly, the in vivo model described herein could be leveraged to develop targeted therapies for severe lung disease.

## Introduction

Chronic obstructive pulmonary disease (COPD) is a chronic inflammatory lung disease and is the third-leading cause of death worldwide (1–3). Primary causes are associated with inhaled substances, such as tobacco smoke, that evoke recruitment of inflammatory cells into the airways (4). Chronic airway inflammation leads to a multitude of remodeling events, including alveolar destruction (emphysema), that conspire to cause airflow obstruction and, ultimately, respiratory failure (5). Importantly, airflow obstruction and the rate of COPD progression are linked to the infiltration of immune cells, in which neutrophils (PMNs) play a prominent role. PMNs release proteases that degrade collagen and elastin. A cornerstone in COPD airway inflammation is the idea that an imbalance occurs between immune cell–derived proteases and endogenous antiproteases in the lung (6, 7). This imbalance leads to unchecked protease activity, resulting in proteolytic destruction of lung extracellular matrix (ECM) and death of airway cells due to loss of ECM survival cues (anoikis) (8–10), which can then progress to enlargement of terminal airspaces (emphysema). A primary player in this ECM destruction is the PMN-derived protease NE, and in patients with COPD and animal models, an imbalance between NE and its antiprotease α-1 antitrypsin (α-1AT) can result in COPD (6, 11–15). While a hereditary form of α-1AT deficiency (16, 17) can result in COPD in the absence of smoking, the vast majority of individuals with COPD have been shown to have adequate α-1AT levels, and it has been unclear how NE in these patients has been able to evade antiprotease inactivation (18, 19).

We and others have shown that exosomes (20–22), small extracellular vesicles (EVs) between 50 and 150 nm, are released by most, if not all, eukaryotic cells, and can harbor proteolytic enzymes on their surface (23–25). Such EVs are increasingly recognized as having a role in lung health, disease, and therapy (26, 27). While investigating exosomes released from PMNs, we identified proteolytically active NE on their surface, along with the PMN marker CD66b (28). Only exosomes termed “activated” (from stimulated PMNs that are degranulating, as opposed to nondegranulating, unstimulated, or “quiescent,” PMNs) contain surface-exposed NE. These activated exosomes acquire NE extracellularly in an α-1AT–resistant orientation and can provoke a COPD-like phenotype in a murine intratracheal (i.t.) transfer model, whereas quiescent exosomes do not.

Importantly, these PMN-derived (CD66b^+^) NE^+^ exosomes are found in the bronchoalveolar lavage fluid (BALF) of COPD patients, but not healthy non-smoking controls, and can provoke an NE-dependent COPD-like phenotype in the mouse i.t. transfer model. The evidence suggests that PMN derived CD66b^+^NE^+^, α-1AT–resistant exosomes may be driving the ECM destruction and alveolar enlargement seen in COPD. Consequently, COPD patients harboring NE^+^ PMN–derived exosomes might be viewed as functionally α-1AT deficient, which, in part, could account for the disease.

Current treatments for COPD do not address the pathogenic role of EVs in promoting emphysema and ECM remodeling, highlighting the need for in vivo models of EV-induced emphysema. Therefore, to provide a way to expand on our discoveries regarding neutrophil-derived EVs and further explore discrete disease-related mechanisms, we developed a mouse-to-mouse EV transfer model. In this study, using an in vivo model of LPS-driven PMN activation and inflammation, we comprehensively studied the characteristics and pathogenicity of the neutrophil-derived EVs associated with the tissue trauma. Our study provides a preclinical in vivo model that allows expansion of the role of airway EVs in the pathogenesis of acute and chronic lung disease, and could provide a platform for novel understanding of disease and therapeutic testing.

## Results

### Acute LPS-driven lung inflammation generates NE-rich EVs.

**Figure 1:**
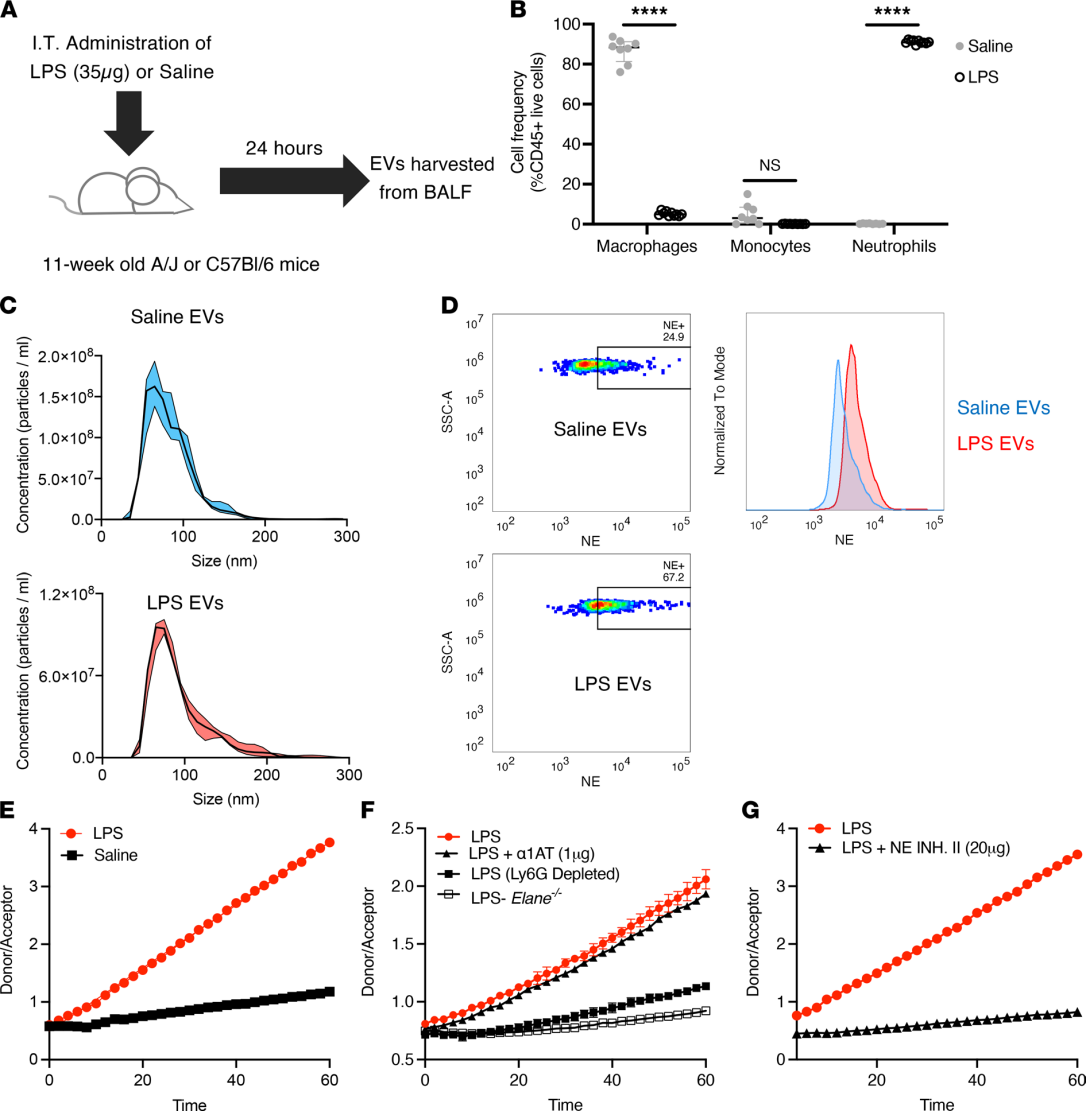
Characterization of airway EVs following i.t. treatment with LPS. (**A**) EVs were harvested from BALF 24 hours following saline or LPS (35 μg) treatment of 11-week-old A/J mice (*n* = 10 per group). (**B**) Flow cytometric analysis of the cellular composition of BALF following LPS or saline treatment. (**C**) Size distribution of airway EVs (LPS and saline treated) determined using NanoSight measurements following purification via differential ultracentrifugation. (**D**) Quantification of surface NE on airway EVs of LPS- and saline-treated mice by bead-based flow cytometric analysis. (**E**) Analysis of NE activity of airway EVs (LPS and saline) using an NE-specific FRET assay. (**F**) NE activity for EVs from LPS-treated *Elane^–/–^* mice or WT mice treated with α-1AT (1 μg) or depleted of Ly6G^+^ EVs. (**G**) NE activity for LPS EVs from **E** pretreated with NE Inhibitor II (NE INH. II; 20 μM) prior to the assay. Data are shown as median and IQR (*n* = 3 replicates per experiment). Statistical analyses were performed using Wilcoxon’s signed-rank test; ****P < 0.0001.

To model the role of neutrophil-derived EVs in generating lung emphysema, we treated mice i.t. with LPS (or sterile saline) (Figure 1A), which resulted in robust neutrophil-dominated acute lung inflammation (Figure 1B and Supplemental Figure 1; supplemental material available online with this article; https://doi.org/10.1172/jci.insight.153560DS1). Characterization of airway EVs isolated from BALF of mice treated with saline or LPS showed that the majority of these vesicles were within the exosome size range of 50–100 nm (Figure 1C) and that the presence of acute neutrophilic inflammation changed neither the size distribution compared with that of the saline controls nor the total number of EVs in the airways (Supplemental Figure 2). Next, given the differential presence of inflammatory cells in the LPS compared with the saline control group, we investigated whether the EVs carried surface-bound NE. Flow cytometry phenotyping of airway EVs showed that LPS-derived EVs carried abundant NE (67.2%) compared with controls (24.9%) (Figure 1D). While saline control EVs carried some NE, likely from inflammation arising from i.t. instillation of saline, NE staining was not detected on *Elane^–/–^* (NE-KO) EVs or non-neutrophil-derived EVs (Supplemental Figure 3). Next, we determined whether increased expression of NE on LPS-derived EVs was accompanied by enhanced proteolytic activity. Fluorescence resonance energy transfer (FRET) assay of the EV-associated NE showed that enzymatic activity was dramatically upregulated in the LPS-treated EV group (Figure 1E). Further, we observed that this increase in activity was dependent both on the presence of NE (relative to its absence in *Elane^–/–^* EVs) and neutrophil-derived EVs (relative to Ly6G depletion) (Figure 1F). We previously showed that EV-associated NE in humans is not subject to inhibition by tissue antiproteases, such as α-1AT (28). Therefore, we investigated whether this feature was conserved across species. As observed in the human specimens, mouse EV-associated NE was not sensitive to α-1AT inhibition (Figure 1F); however, NE activity was inhibited by a small molecule inhibitor of NE (Figure 1G). These results demonstrate that neutrophil-derived EVs generated during lung inflammation exhibit increased α-1AT–resistant NE activity, thus warranting more extensive studies into whether they can elicit tissue remodeling in vivo. Collectively, these results also demonstrate that activated EVs from mouse PMNs are similar, if not identical, to those from human PMNs in terms of harboring α-1AT–resistant NE on a Ly6G^+^ EV population.

### LPS-derived EVs induce lung damage and emphysema.

To determine whether LPS-derived EVs are pathogenic and, if so, the optimal conditions under which to model airway damage mediated by EVs, we delivered saline- and LPS-derived EVs i.t. for 1 week at a single dose or over 3 doses 2 days apart (Supplemental Figure 4). Airway damage was quantified by alveolar enlargement (mean linear intercepts [*L_m_*]). The dosing strategies resulted in similar levels of alveolar enlargement after 1 week in the LPS EV group when compared with the saline EV control group, with LPS-derived EVs inducing significantly more damage. Therefore, to minimize the invasiveness of the procedure, the regimen selected for the remainder of the study was 1 dose of EVs followed by tissue analysis 1 week later.

**Figure 2:**
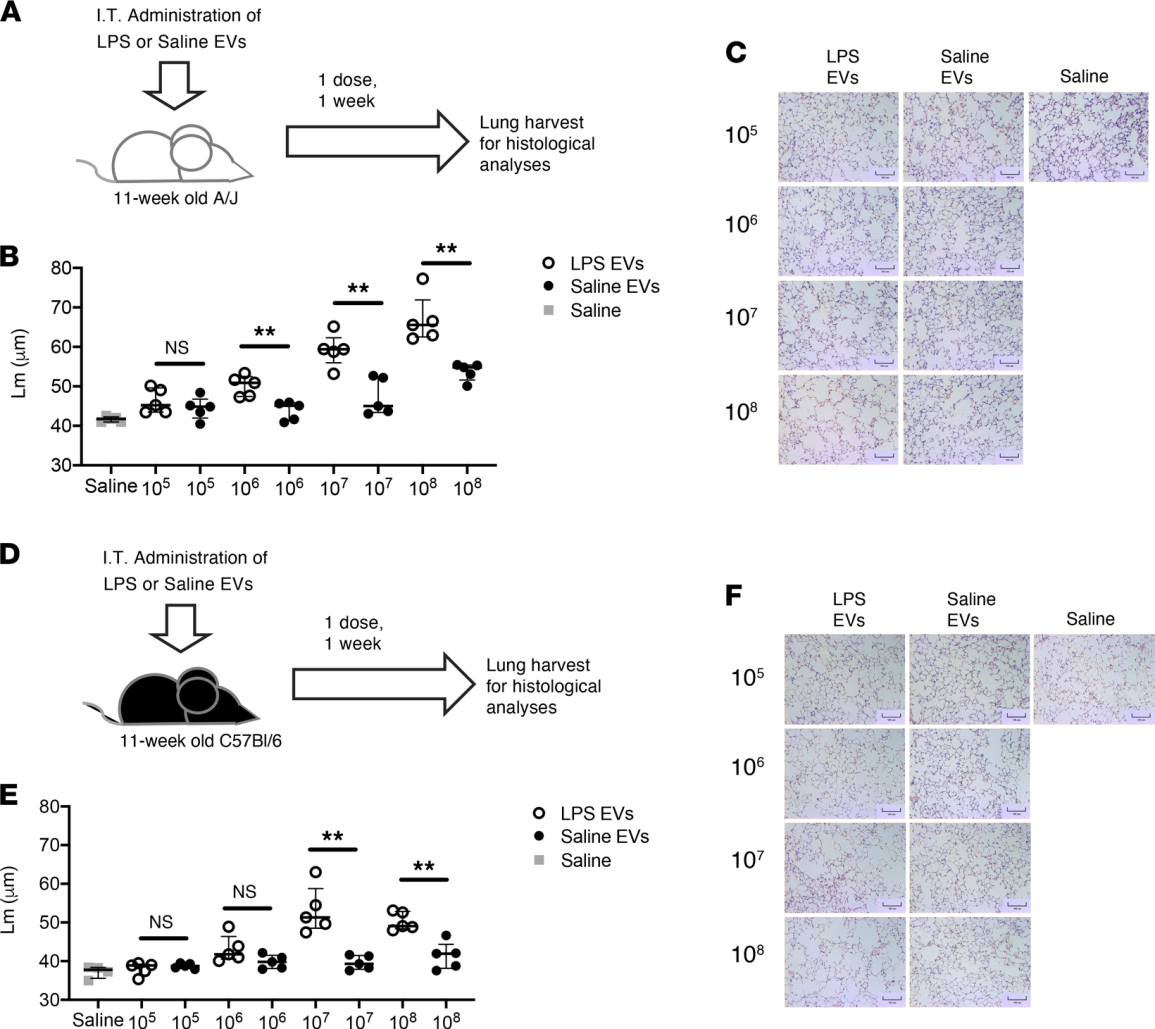
LPS-treated airway EVs induce alveolar damage in naive recipient mice. (**A**) EVs were transferred i.t. into 11-week-old female A/J mice. Mice received 10^8^, 10^7^, 10^6^, or 10^5^ EVs in a single dose over 1 week. (**B**) *L_m_* were quantified 1 week from the initial treatment (*n* = 5 per group). (**C**) Representative images (H&E) of EV-treated mice (LPS and saline). Scale bars: 100 μm. (**D**) EVs were transferred i.t. into 11-week-old female C57BL/6 mice. Mice received 10^8^, 10^7^, 10^6^, or 10^5^ EVs in a single dose over 1 week. (**E**) *L_m_* were quantified 1 week from the initial treatment (*n* = 5 per group). (**F**) Representative images (H&E) of EV-treated mice (LPS and saline). Scale bars: 100 μm. Data are shown as median and IQR (representative of 2 independent experiments). Statistical analyses were performed using Wilcoxon’s signed-rank test; **P < 0.01.

**Figure 3:**
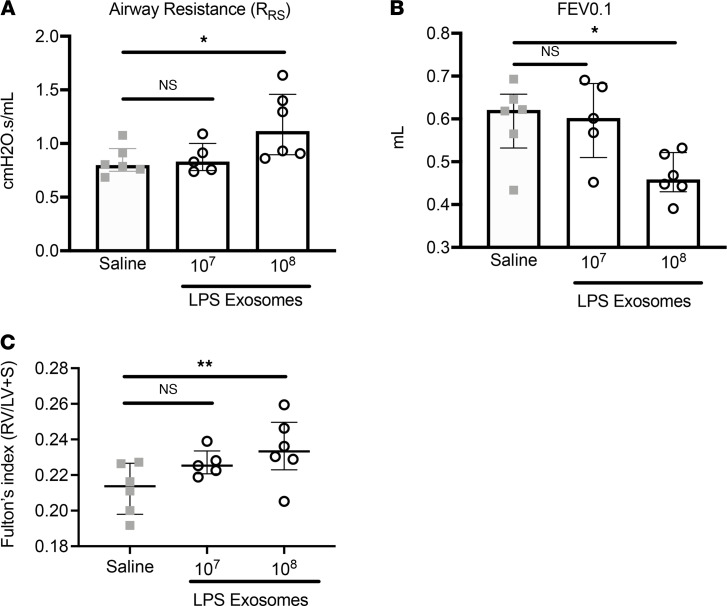
Systemic and airway physiology measurement after exosome delivery. WT C57BL/6 mice were treated with LPS exosomes for 1 week (*n* = 5 per group). Airway physiology was determined using flexiVent for measures of airway resistance (**A**) and FEV0.1 (**B**). (**C**) The systemic impact of exosome delivery were measured by Fulton’s index. Statistical analyses were performed using Wilcoxon’s signed-rank test; *P < 0.05, **P < 0.01. Data are shown as median and IQR (representative of *n* = 2 independent experiments).

Next, we sought to determine the optimal number of EVs required to elicit airway enlargement. A/J mice were treated with increasing numbers of EVs (Figure 2A), and airway damage was quantified after 1 week (Figure 2B). The results showed a dose-response relationship for LPS-derived EVs, with statistically significant alveolar enlargement observed with 1 × 10^6^ EVs and a robust response beginning at 1 × 10^7^ EVs (Figure 2, B and C). The same trend was present in age-matched C57BL/6 mice (Figure 2, D–F), suggesting that the mechanism of EV-mediated alveolar enlargement is conserved across strains. Furthermore, we observed that the EV-mediated alveolar enlargement from a single 1 × 10^7^ EV dose lasted for at least 3 weeks (Supplemental Figure 5, A–C), with the induction of low levels of inflammation at weeks 1 and 2 (Supplemental Figure 5, D–G). The degree of emphysema at week 3 was lower than at weeks 1 and 2; however, the difference was not significant, suggesting a long-lasting effect independent of the presence of high degree of inflammation. We also determined whether the alveolar enlargement in the LPS exosome groups observed by histological analysis translated into local and systemic pathological changes. Mice treated with 1 × 10^8^ LPS exosomes showed increased airway resistance, lower forced expiratory volume in 0.1 seconds (FEV0.1), and right-ventricular hypertrophy 1 week after the first dose (Figure 3, A–C). The latter effect continued to trend upward, albeit insignificantly, when mice were treated with 1 × 10^7^ LPS exosomes, suggesting a possible threshold effect. Together these data show that local changes mediated by LPS-activated airway EVs can translate into systemic pathological changes characteristic of emphysema in a short period of time.

### LPS-derived EVs promote alveolar enlargement in a PMN- and NE-dependent manner.

**Figure 4:**
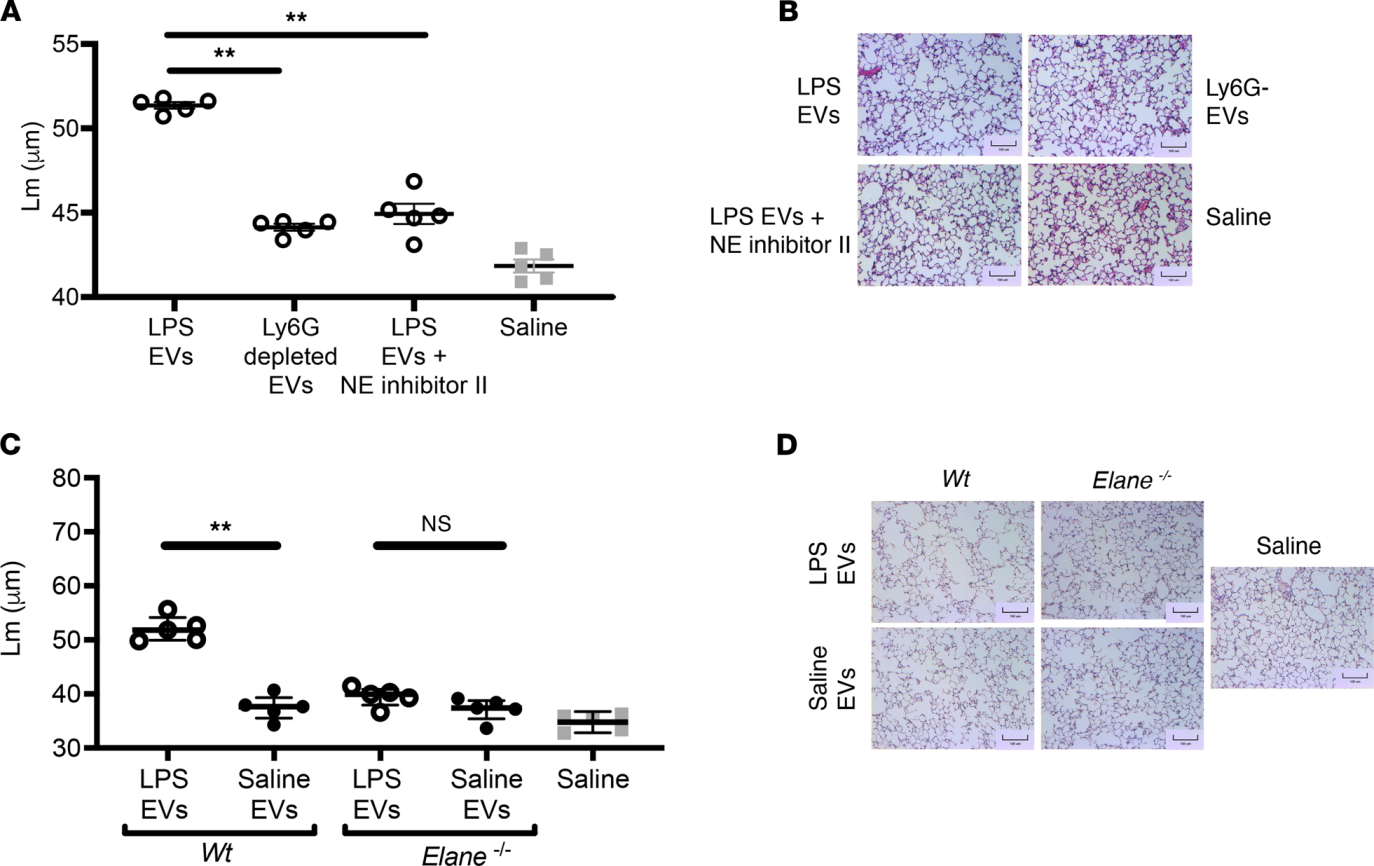
LPS EV–mediated lung is driven by neutrophil EVs bearing neutrophil elastase. (**A**) Eleven-week-old female C57BL/6 mice received a single dose of 1 × 10^7^ EVs i.t. from either WT or *Elane^–/–^* mice treated with saline or LPS (35 μg). *L_m_* were quantified 1 week from the initial treatment (*n* = 5 per group). (**B**) Representative images (H&E) of EV-treated mice. Scale bars: 100 μm. (**C**) Eleven-week-old female C57BL/6 mice received single dose of 1 × 10^7^ of LPS-treated EVs i.t. with or without Ly6G depletion to remove neutrophil-derived EVs. *L_m_* were quantified 1 week from the initial treatment (*n* = 5 per group). (**D**) Representative images (H&E) of EV-treated mice. Scale bars: 100 μm. Data are shown as median and IQR (*n* = 1 experiment). Statistical analyses were performed using Wilcoxon’s signed-rank test; **P < 0.01.

Lung-derived EVs generated in response to LPS showed markedly increased NE activity and caused alveolar enlargement. Consequently, we investigated whether the pathogenic effect of these EVs was dependent solely on neutrophil-derived EVs via an NE-dependent mechanism. To test this, we either depleted LPS-derived EVs of the neutrophil-derived population via a bead-based pulldown method (Ly6G^+^) or treated them with a small molecule inhibitor for NE. The EVs were then instilled i.t. into naive mice, and lungs were harvested for histological analysis 1 week later. Notably, depletion of neutrophil-derived EVs drastically reduced the EV-mediated alveolar enlargement (Figure 4, A and B). Furthermore, inhibition of EV-associated NE mediated a similar reduction in *L_m_*, suggesting that the mechanisms of airway pathology are mediated by NE present on neutrophil-derived EVs. To further test this observation, we isolated EVs from WT or NE-KO (*Elane^–/–^*) mice after induction of airway inflammation via LPS or saline control. As expected, LPS-derived airway EVs from WT mice promoted alveolar enlargement. In stark contrast, this effect was almost completely abrogated in the absence of NE (Figure 4, C and D). Further, the low level of neutrophil inflammation in the airways was also abrogated when neutrophil EVs were absent (Ly6G-depleted) or in the absence of NE or NE activity (Supplemental Figure 6). Together, these data further confirm a pathological NE-EV–dependent mechanism.

## Discussion

Neutrophil migration into the airways of patients with emphysema and COPD has been well documented (29); however, the mechanisms influencing their impact on disease severity and progression remains poorly understood. Several soluble mediators, such as reactive oxygen species, inflammatory cytokines, and proteases have been linked to the inflammatory responses in emphysema (30), likely via induction of epithelial necroptosis (31). We previously showed that tissue injury in COPD could also be mediated by neutrophil-derived exosomes isolated from human specimens via an NE-mediated mechanism (28).

Several in vivo models have been developed to study COPD, including cigarette smoke–induced (32), elastase-induced (33), and LPS-induced emphysema (34, 35), or a combination of these (36). However, mechanisms of disease progression remain elusive, thus slowing progression of the development of novel therapeutics. This study introduces a methodology to address the role of EV-induced pathology. Interestingly, while LPS administration can induce emphysema (37), administration of LPS-derived neutrophil EVs results in a much faster progression to alveolar enlargement with a 500-fold-lower dose, suggesting the presence of LPS-induced protective mechanisms in donor mice that is absent in non-LPS-treated recipients. Further, the identification of LPS-induced neutrophil-derived proteolytic EVs provides a context for study of the impact of these entities during bacteria-driven COPD exacerbations. Future studies should consider models of bacterial challenge with smoke-induced emphysema to determine whether these EVs are more prominent and may provide progressive tissue remodeling mediated by NE that would translate in changes in airway physiology.

Our results show that, although there is not a robust T cell signature in this acute inflammatory model of tissue injury, neutrophils are present at low levels in the BALF of neutrophil-derived EV–treated mice. Further, while our results demonstrate a role of neutrophil-derived EVs in promoting alveolar enlargement, EV contributions from other resident cells during emphysema cannot be excluded, warranting further, comprehensive investigation into the role that EVs play in the pathogenic response.

In resolving inflammatory responses, NE activity is counteracted by tissue antiproteases such as elafin and α-1AT (38, 39). However, in pathologic conditions, such as emphysema and COPD, protease/antiprotease imbalances have been described (40–42), suggesting an unopposed enzymatic activity leading to tissue damage and remodeling. To this end, elastase inhibitors have been developed and used in clinical trials for COPD (43), but with little to no influence on disease progression (44, 45). While these clinical studies show little improvement of patient outcomes, one must consider the route of administration of such therapies. Indeed, the data presented in this study show that lung damage in emphysema and COPD is mediated by EV-bound NE present in the airways, which confers resistance to tissue antiproteases. Therefore, the failure of these small molecule inhibitors may be reversed by the development of an airway delivery system for these therapeutics, which are active not only on free NE, but on the EV-bound form as well. Of course, one also needs to consider the possibility of reduced potency of a given small molecule inhibitor against EV-associated NE compared with the solution-phase NE for which they were developed.

This study describes the development of a model of lung injury via mouse-to-mouse transfer, including evidence for sustained injury for weeks after initial EV administration. Importantly, this is a rapid model that mimics key features of COPD and may serve as a robust system for preclinical drug studies related to attenuating tissue remodeling and emphysema. In conclusion, this study highlights a rapid, neutrophil-driven mechanism of emphysema mediated by mouse neutrophil–derived EV–bound NE, which recapitulated with fidelity what was observed with human neutrophils (28). Broadly, this study establishes a robust in vivo model to study the mechanisms underlying disease progression in COPD, opening opportunities to develop and validate new targeted therapeutics.

## Methods

### Mouse models.

All experiments were carried out in 11-week-old female mice. A/J and C57BL/6, and mice were purchased from the Jackson Laboratory at 10 weeks of age and rested for 1 week prior to use. *Elane^–/–^* mice were purchased from the Jackson Laboratory (B6.129X1-*Elane^tm1Sds^*/J), and female mice were used at 11 weeks of age.

### Mouse EV generation.

Eleven-week-old female mice were treated i.t. with 50 μL sterile saline (0.9%) or 35 μg *Pseudomonas aeruginosa*–derived LPS diluted in 50 μL sterile saline (MilliporeSigma). BAL was performed 24 hours after treatment with sterile PBS and pooled for each group.

### Mouse EV isolation and characterization.

BAL were spun at 500*g* for 10 minutes at 4°C to remove airway cells. The supernatants were sequentially centrifuged at 10,000*g*, 4°C for 1 hour, followed by a 100,000*g* ultracentrifugation for 2 hours. Pelleted EVs were resuspended in ultracentrifuged PBS, then characterized and quantified using a NanoSight N3000 (Malvern Panalytical).

### EV delivery.

Dose-response assays were performed using 10^9^, 10^8^, 10^7^, 10^6^, or 10^5^ EVs diluted in 50 μL sterile saline. Mice were anesthetized with isoflurane, and EVs were delivered i.t. either at a single dose or 3 doses (every other day) for the duration of 1 week. All other assays were carried out with a single dose of 1 × 10^7^ EVs over 1 week.

### Ly6G EV depletion.

Streptavidin-coated magnetic beads (Spherotech) were washed in ultracentrifuged PBS, and 5 μL beads (1% w/v) were incubated with 300 ng biotinylated anti–mouse Ly6G antibody (Miltenyi Biotec) for 2 hours on ice. Antibody-coated beads were then washed 3 times with ultracentrifuged PBS and incubated with 1 × 10^10^ LPS-derived airway EVs overnight at 4°C on an end-over-end rotating wheel. The next day, beads were removed by means of a magnetic stand, and free EVs were quantified using a NanoSight N3000 (Malvern Panalytical).

### Histology.

Mice were sedated with pentobarbital (50 mg/kg). The lung circulation was infused with sterile PBS to remove blood cells, then the lungs were fixed isobarically using 10% buffered formalin (MilliporeSigma) delivered i.t. Lungs were excised and transferred in 10% buffered formalin for 48 hours. The left lungs were embedded in paraffin, sectioned, and stained with H&E for morphometric analysis.

### Lung morphometric analysis.

H&E-stained left lung sections were imaged using an INFINITY2 photomicrographer (Lumenera Corp.). *L_m_* were quantified using the INFINITY ANALYZE2-1 program. Briefly, 4 nonconsecutive sections per mouse were imaged at 10× magnification and overlaid with a 100 mm^2^ grid. Photomicrographs (3 per section) were analyzed for the number of intersections of 3 noncontiguous alveolar septae with the walls of 100 mm^2^ grid. *L_m_* for each image were quantified by dividing the number of intercepts for each square by 400 mm length. The final *L_m_* was obtained by averaging the 36 counts per each lung across each 400 mm measurement.

### Flow cytometry.

Airway cells obtained from the BAL were resuspended in cold PBS-EDTA (2.5 mM) and incubated for 10 minutes on ice in the dark with a mouse Fc block (BioLegend) and the Zombie Near-IR Live/Dead stain (BioLegend). After the preincubation, cells were stained for 20 minutes on ice in the dark for surface expression of CD45 (clone 30-F11), CD11c (clone N418), Ly6G (clone 1A8), Ly6C (clone HK1.4), CD11b (clone M1/70), CD4 (clone GK1.5), CD8 (clone 53-6.7), NK1.1 (clone S17016D) and CD3 (clone 145-2C11) (all antibodies were purchased from BioLegend). Cells were then washed 3 times with cold PBS-EDTA and then fixed overnight at 4°C with the Lyse/Fix Phosflow (BD Biosciences). The next day, the fixative was removed following a 500*g*, 4°C centrifugation for 10 minutes, and cells were resuspended in cold PBS-EDTA. Cell acquisition was performed on a BD FACS Symphony (BD Biosciences), and data were analyzed using FlowJo v10.7 (BD Biosciences).

### EV flow cytometry.

Streptavidin-coated magnetic beads (Spherotech) were washed in ultracentrifuged PBS, and 5 μL beads (1% w/v) were incubated with 300 ng of biotinylated anti–mouse Ly6G antibody (Miltenyi Biotec, clone REA526) for 2 hours on ice. Antibody-coated beads were then washed 3 times with ultracentrifuged PBS and incubated with 1 × 10^8^ EVs overnight at 4°C on an end-over-end rotating wheel. The next day, beads were washed 3 times with ultracentrifuged PBS and incubated with 1 μg mouse Fc block (BioLegend) for 15 minutes on ice, followed by a 30-minute incubation in the dark on ice with 1 μg anti–mouse NE antibody (R&D Systems, clone 887105) conjugated with APC (Lightning-link, Novus Biologicals). Bead-EV conjugates were washed 3 times with ultracentrifuged PBS before acquisition on a Cytoflex cytometer (Beckman Coulter).

### Neutrophil elastase activity.

Neutrophil elastase activity was measured using a FRET probe (Sirius Fine Chemicals). 1 × 10^10^ EVs were incubated in the activation buffer (100 mM Tris-HCl, 500 mM NaCl, pH 7.5) with 1 μM of the Nemo-1 probe at 37°C. Fluorescence (excitation, 354 nm) was measured every 2 minutes for a total of 30 minutes (donor: 400 nm; acceptor: 490 nm). Activity was quantified using the ratio of donor/acceptor fluorescence measurements.

Inhibition of NE was performed with NE Inhibitor II (20 μM, EMD Millipore) or with recombinant mouse SERPINA1 (α-1AT, 1 μg; Ls Bio). EVs were preincubated with the inhibitors for 30 minutes at 37°C prior to activity measurement.

### Physiological measurements of lung function.

One week after treatment with LPS- and saline-derived airway EVs, mice underwent pulmonary function testing using a flexiVent apparatus (SCIREQ) with the Negative Pressure Forced Expiration (NFPE) extension. All data were analyzed using flexiWare 8.0 software. System and tube calibrations were completed prior to the experiment, and subsequent tube calibrations were performed before testing of each new subject. Following anesthetization with ketamine/xylazine, a tracheostomy was performed on each mouse using a 20G Angiocath, and mice were connected to the flexiVent apparatus. The system delivered a tidal volume of 6 mL/kg, with a respiratory rate of 150/min. Pulmonary measurements, including airway resistance (R), compliance (C), elastance (E), and FEV0.1, were taken in triplicate by the software using multiple respiratory perturbation programs. All pulmonary parameters were recorded and presented as an average of 3 independent measurements.

### Statistics.

All analyses were performed using nonparametric statistics as detailed in the figure legends. Data are shown as median and IQR, and significance threshold was set at α = 0.05.

### Study approval.

All animal studies and protocols were approved by the IACUC at the University of Alabama at Birmingham.

## Author contributions

JEB, CM, MCM, and AG conceived the study and wrote the manuscript; CM, MCM, KRG, DWR, and LV carried out the experiments.

## Supplementary Material

Supplemental data

## Version 1 01/25/2022

In-Press Preview

## Version 2 02/22/2022

Electronic publication
