# Supplementary material for: An in vivo model for extracellular vesicle–induced emphysema
Source: JCI Insight. 2022 Jan 25;7(4):e153560. doi: 10.1172/jci.insight.153560 (PMC8876451; doi:10.1172/jci.insight.153560)
Supplement: Supplemental data [file jciinsight-7-153560-s005.pdf]

JCI insight – Research article

An in vivo model for extracellular vesicle-induced emphysema

Margaroli C.[1,2,†], Madison M.C.[1,2,†], Viera L.[1], Russell D.W. [1,2],

Gaggar A.[1-4*], Genschmer K.R.[1,2#], Blalock J.E. [1,3#]

Figures: 6

Supplemental Figures

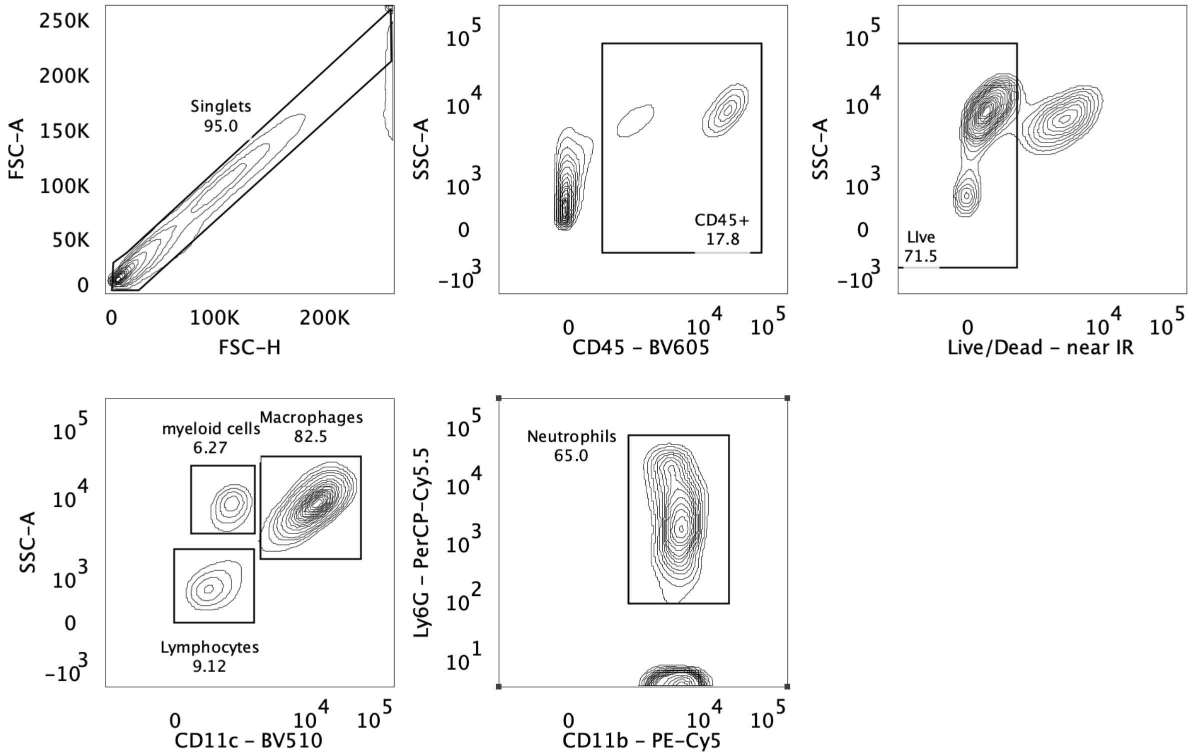

Figure S1. Flow cytometry gating strategy. Airway cells were isolated from BAL of saline or LPS-treated mice and analyzed by flow cytometry. Live neutrophils were identified as SSC-A^{hi} CD11c^- Ly6G^+ CD11b^+, macrophages as SSC-A^{hi} CD11c^+ Ly6G^-, and lymphocytes as SSC-A^{lo} CD11c^- Ly6G^-.

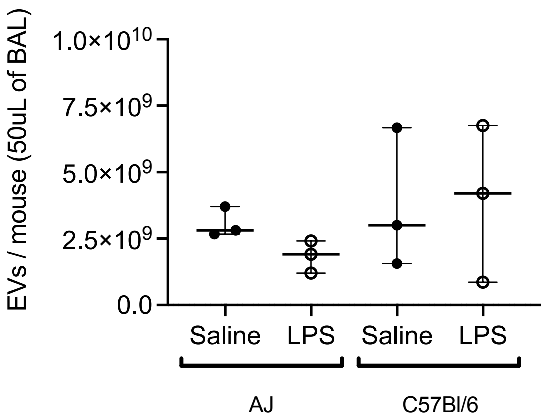

Figure S2. Quantification of airway EVs. EVs were harvested from BAL fluid 24 hours following saline or LPS (35ug) treatment of 11-week-old A/J mice or C57Bl/6 mice. Data are shown as median and interquartile range (n=3 independent experiments). Statistical analyses were performed using Wilcoxon signed-rank test, ns = not significant.

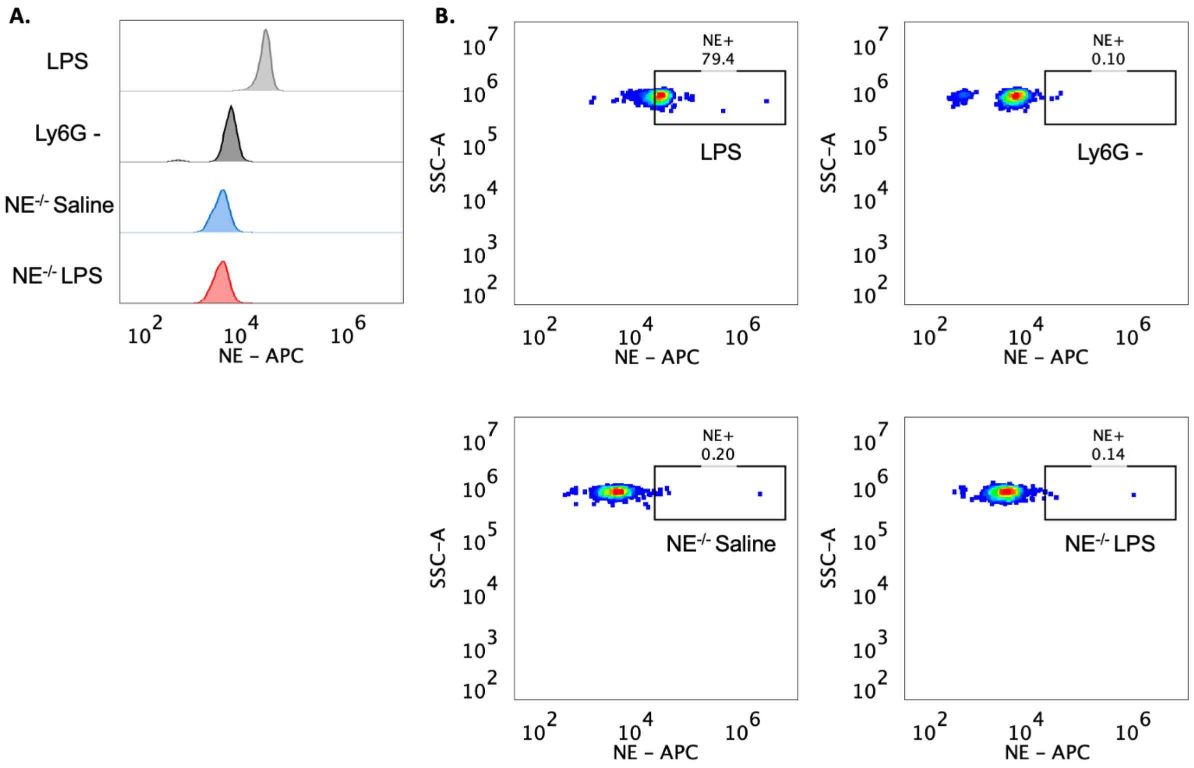

Figure S3. Characterization of surface NE on mouse EVs. (A) Surface NE quantification on mouse airway EVs by bead-based flow cytometric analysis. From top to bottom: LPS treated wild type mice - CD63 EV pull-down (grey); LPS treated wild type mice - CD63 EV pull-down after Ly6G+ EV depletion (black); saline treated $NE^{-/-}$ mice - Ly6G pulldown (blue); LPS treated $NE^{-/-}$ mice - Ly6G pulldown (red). **(B)** Quantification of NE+ EVs for the groups described in (A).

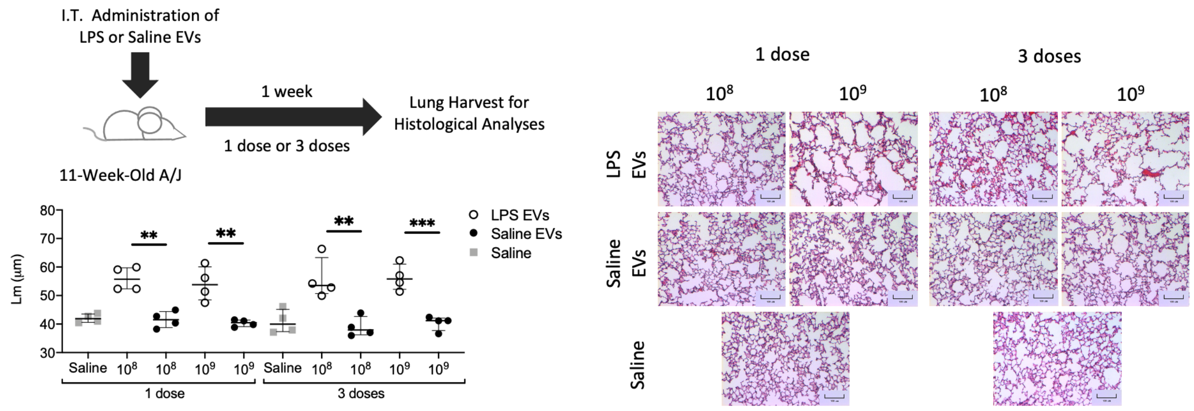

Figure S4. Dosing strategy for saline or LPS intratracheal EVs delivery. EVs were transferred intratracheally to 11-week old female A/J mice. Mice received 10^9 or 10^8 exosomes in either a single dose or over 3 doses for the duration of 1 week. Mean Linear Intercepts (L_m) were quantified one week from the initial treatment as a measure of alveolar enlargement (n=4 per group). Representative images (H&E) of EVs-treated mice (LPS and saline). Scale bars represent 100 μm. Data are shown as median and interquartile range (n=1 experiment). Statistical analyses were performed using Wilcoxon signed-rank test, ** $p < 0.01$, *** $p < 0.001$, ns = not significant.

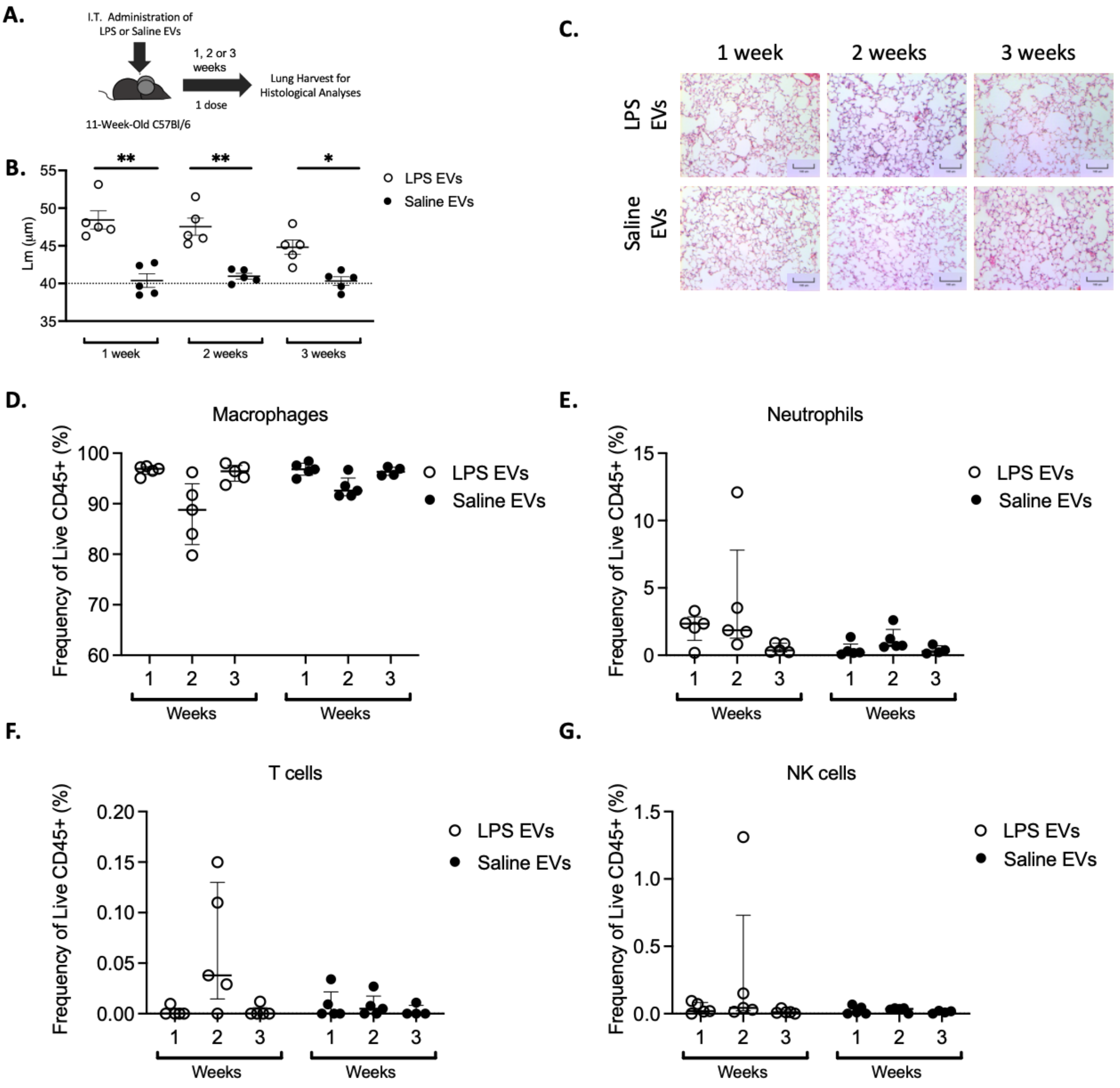

Figure S5. Time course upon EVs delivery. (A) 10^7 EVs were transferred intratracheally in a single dose to 11-week old female C57BL/6 mice. Lungs were harvested 1 week, 2 weeks and 3 weeks after the initial dose. (**B**) L_m were quantified one week from the initial treatment (n=5 per group). (**C**) Representative images (H&E) of EV-treated mice (LPS and saline). Scale bars represent 100 μm. **(D-G)** Immune cells in the BAL of mice treated with 10^7 Evs were characterized by flow cytometry as defined in Figure S1. T cells were identified as CD3+ and NK cells as NK1.1+. Data are shown as median and interquartile range (n=1 experiment). Statistical analyses were performed using Kruskal-Wallis test, ** $p < 0.01$, * $p < 0.05$.

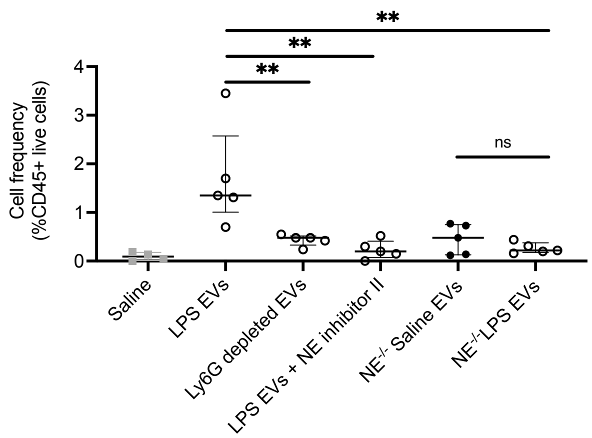

Figure S6. Flow cytometry of BAL after EV delivery. Neutrophils were quantified by flow cytometry in the BAL of mice treated with one 10^7 dose of saline or LPS EVs from WT or NE KO mice (n=5 per group). Data are shown as median and interquartile range (n=1 experiment). Statistical analysis was performed with Kruskal-Wallis test, ** $p < 0.01$, ns = not significant.
